# Efficacy of cannabis-based medicine in the treatment of Tourette syndrome: a systematic review and meta-analysis

**DOI:** 10.1007/s00228-024-03710-9

**Published:** 2024-07-10

**Authors:** Ibrahim Serag, Mona Mahmoud Elsakka, Mostafa Hossam El din Moawad, Hossam Tharwat Ali, Khalid Sarhan, Sally Shayeb, Islam Nadim, Mohamed Abouzid

**Affiliations:** 1 Faculty of Medicine, Mansoura University, Mansoura, Egypt; 2 Faculty of Pharmacy, Damanhour University, Damanhour, Egypt; 3 Alexandria Main University Hospital, Alexandria, Egypt; 4 Faculty of Medicine, Suez Canal University, Ismailia, Egypt; 5 Qena Faculty of Medicine, South Valley University, Qena, Egypt; 6 Faculty of Public Health, Al-Quds University, Jerusalem, Palestine; 7 Faculty of Medicine, Kafrelsheikh University, Kafrelsheikh, Egypt; 8 Department of Physical Pharmacy and Pharmacokinetics, Faculty of Pharmacy, Poznan University of Medical Sciences, Rokietnicka 3 St., 60-806 Poznan, Poland; 9 Doctoral School, Poznan University of Medical Sciences, 60-812 Poznan, Poland

**Keywords:** Tourette syndrome, Cannabis, TS

## Abstract

**Background:**

Tourette syndrome (TS) is a neurodevelopmental disorder characterized by motor and phonic tics. It is a condition that affects between 0.3% and 0.7% of children, and its pathophysiology remains largely elusive. TS is associated with structural and functional alterations in corticostriatal circuits and neurochemical imbalances. Even though TS is currently incurable, there are established treatment options available, including behavioral therapy and neuroleptics. The use of cannabis-based medicine for tic management is an emerging therapeutic strategy, although its efficacy is still under investigation. It is hypothesized to interact with the endogenous cannabinoid system, but further research is required to ascertain its safety and effectiveness in TS.

**Aim:**

In our systematic review and meta-analysis, we aim to assess the effectiveness of cannabis-based medicine in the treatment of TS.

**Methods:**

We searched PubMed, Cochrane, Scopus, and Web of Sciences until February 2024. We included clinical trials and cohort studies investigating the efficacy of cannabis-based medicine in the treatment of TS. Data extraction focused on baseline characteristics of the included studies and efficacy outcomes, including scores on the Yale Global Tic Severity Scale (YGTSS), Premonitory Urge for Tics Scale (PUTS), and Yale-Brown Obsessive Compulsive Scale (Y-BOCS). We conducted the meta-analysis using Review Manager version 5.4. software. We compared the measurements before and after drug intake using mean difference (MD) and 95% confidence interval (CI).

**Results:**

In total, 357 articles were identified for screening, with nine studies included in the systematic review and 3 in the meta-analysis. These studies involved 401 adult patients with TS treated with cannabis. YGTSS revealed a significant reduction in total scores (MD = -23.71, 95% CI [-43.86 to -3.55], *P* = 0.02), PUTS revealed a significant decrease in scores (MD = -5.36, 95% CI [-8.46 to -2.27], *P* = 0.0007), and Y-BOCS revealed no significant difference in score reduction (MD = -6.22, 95% CI [-12.68 to 0.23], *P* = 0.06).

**Conclusion:**

The current study indicates promising and potentially effective outcomes with the use of cannabis-based medicine in mitigating the severity of tics and premonitory urges. However, there is a need for larger, placebo-controlled studies with more representative samples to validate these findings.

**Supplementary Information:**

The online version contains supplementary material available at 10.1007/s00228-024-03710-9.

## Introduction

Tourette syndrome (TS), named after Gilles de la Tourette in 1885, is a neurodevelopmental and behavioral disorder characterized by motor and phonic tics [1–3]. The American Psychiatric Association Diagnostic and Statistical Manual of Mental Disorders defines TS as the presence of chronic motor and phonic tics for at least a year starting before the age of 18 years after excluding possible secondary causes [4]. Most studies estimated the prevalence of TS to be between 0.3 and 0.7% among children and adolescents [5–8]. Nevertheless, experts agree that the condition is usually underdiagnosed with a long duration between the onset of symptoms and diagnosis [9].

The pathophysiology of TS is not entirely understood. However, structural and functional changes in multiple corticostriatal circuits may account for the behavioral manifestations. Involvement of the neurochemical disbalance in the dopaminergic system has also been proposed [10, 11]. TS is a heterogeneous condition in terms of severity of symptoms and comorbidities; fortunately, severe TS is rare. Patients variably become tolerant to tics socially and functionally [9]. Attention deficit hyperactive disorder (ADHD) is the most common comorbidity with TS, co-existing in around 60% of cases and followed by obsessive–compulsive disorder (OCD), and can be disabling more than the tics [10].

Although there is no cure for TS, patients can benefit from controlling tics [12, 13]. Notably, many patients with tics that do not interfere with their lives do not require management. Medications and behavioral management can be provided if tics cause stress, injuries, or interference with daily life [10, 12–14]. Behavioral therapy has demonstrated promising results in reducing the severity of tics and thus can be considered the first-line therapy for TS [10, 12]. On the other hand, patients respond variably to medications. Alpha-2 adrenergic agonists, such as clonidine and guanfacine, are frequently used as a first-line pharmacological approach for suppressing tics associated with TS [15]. Notably, for patients with co-existing attention deficit hyperactivity disorder (ADHD), these medications offer the potential benefit of reducing both tic severity and ADHD symptoms. Treatment options include clonidine alone, a combination of clonidine and methylphenidate (a stimulant medication for ADHD), or guanfacine [3, 10]. Other medications include neuroleptics, topiramate, and anticholinergics [2, 3, 10, 14].

*Cannabis sativa* has a history of recreational and medicinal use. Its potential therapeutic effects in various conditions have recently gained attention [16]. There are many forms of cannabis-based medicines (CBM), including tetrahydrocannabinol, THX-110 combinations, cannabinoid oils, and synthetic cannabinoids such as palmitoylethanolamide [17]. The human endocannabinoid system (ECS) is a complex signaling system that includes cannabinoid receptors, their endogenous ligands (endocannabinoids), and biosynthetic and hydrolytic enzymes [18]. ECS is crucial in various physiological processes, including cognition, learning, memory, pain perception, gastrointestinal function, and autonomic control [16, 19, 20]. CBM can interact with the ECS by binding to cannabinoid receptors, primarily CB1 and CB2. CB1 receptors are predominantly found in the central nervous system, while CB2 receptors are more abundant in immune cells [16, 19, 21]. This interaction may modulate the ECS's activity and influence various physiological functions. However, the specific mechanisms of CBM are still being investigated [20–30].

Evidence supports the use of medicinal cannabis in conditions such as chronic pain [31, 32], resistant chemotherapy-induced vomiting and nausea [33], and sleep disorders [34, 35]. Their use for controlling tics has recently increased, with many trials and reports showing encouraging results [23–30]. Recent reviews revealed poor-quality evidence and inconclusive results for the efficacy of CBM for neurodevelopmental and movement disorders [20–22].

There is a lack of evidence to recommend to patients for management of TS [14, 36–40]. In this systematic review and meta-analysis, we aimed to provide a comprehensive assessment of the efficacy of CBM in TS by summarizing the totality of evidence to date regarding their efficacy.

## Materials and methods

### Study design

The present systematic review and meta-analysis followed the Preferred Reporting Items for Systematic Reviews and Meta-analyses (PRISMA) statement [41, 42], encompassing the published clinical trials and cohort studies on using CBM for TS.

### Literature search

A systematic literature review of PubMed Medline, Cochrane, Scopus, and Web of Sciences databases was to identify eligible published studies up to February 2024 restricted to the English language. We constructed a thorough search string using the entry terms of relevant keywords (Tourette Syndrome, cannabis, cannabis*based medicine). The published reviews and reference lists of selected papers were also searched up to February 2024.

### Eligibility criteria: types of studies, participants, and intervention

We selected eligible studies based on pre-identified criteria. We included only clinical trials and cohort studies, and our PICOS (Population, Intervention, Comparison, Outcome) was as follows:

- Population: adult patients diagnosed with TS.
- Intervention: treatment with CBM.
- Comparison: placebo or no therapy.
- Outcome: tic severity, premonitory urges, and OCD symptoms were measured by the Yale Global Tic Severity Scale (YGTSS), the Premonitory Urge for Tics Scale (PUTS), and the Yale-Brown Obsessive Compulsive Scale (Y-BOCS), respectively.

Exclusion criteria were review articles, theses, conference abstracts, editorials, commentaries, case reports, articles assessing the efficacy of cannabis in disorders other than TS, and articles written in languages other than English.

### Screening of the studies

Three authors (MME, SS, and IN) independently screened titles and abstracts of the retrieved citations according to the inclusion and exclusion criteria. Consequently, the full text of potentially eligible records was retrieved. Four authors (IS, MME, SS, and IN) independently screened the full-text papers according to the inclusion criteria, with the reconciliation of any differences conducted by the author IS.

### Data extraction and outcome measures

Following screening, we extracted relevant data using specifically designed extraction forms. Three authors (MME, SS, and IN) extracted the data and the author IS resolved any differences. Extracted data included but was not limited to study methodology and design, type of CBM and dose, participants' demographic characteristics and comorbidities, frequency of tics, and main findings. Efficacy outcome measures included scores on the YGTSS, PUTS, and Y-BOCS:

- YGTSS: Lower scores mean less severe tics, assessing severity and frequency [43]
- PUTS: Higher scores indicate more severe premonitory urges before tics [44]
- Y-BOCS: Higher scores represent more severe OCD symptoms, measuring obsessions and compulsions [43]

### Assessment of risk of bias

We have used the Cochrane risk-of-bias 2 tool (RoB 2) [45] for randomized controlled trials (RCTs), whereas, for cohort studies, the Newcastle–Ottawa scale (NOS) [46] was implemented. For each study, two authors independently assessed the risk of bias, and a third author resolved any differences.

### Meta-analysis

We used Review Manager (RevMan) software (version 5.4) for data analysis. Continuous data was presented as the mean difference between pre-treatment and post-treatment with a 95% confidence interval (CI). A *p* value of ≤ 0.05 was deemed statistically significant. The heterogeneity in the data was examined through I-square and *p* value for significance. The Cochrane Handbook’s guidelines for meta-analysis were followed when interpreting the I-square test (0–30% = may not be significant, 30–60% = may represent moderate heterogeneity, 60–90% = may represent substantial heterogeneity, and 75–100% = significant heterogeneity) and a *p* value < 0.05. The random effects model was adopted for a broader, more realistic CI because our data was heterogeneous in some outcomes [47].

## Results

### Search result

**Fig. 1:**
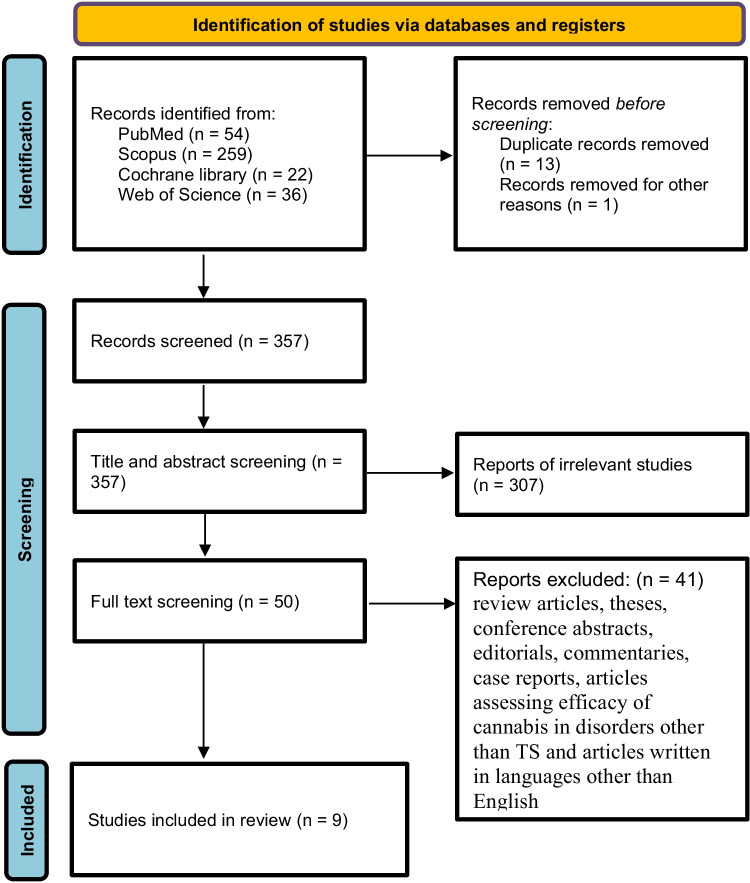
PRISMA flowchart of included studies.

Our search yielded 357 articles. After screening the titles and abstracts, only 50 articles were deemed eligible for full-text screening. Ultimately, nine studies were included in this systematic review, with three of these studies qualifying for the meta-analysis (refer to the PRISMA flow diagram in Fig. 1). The systematic review encompassed a total of 401 adult patients with TS who were treated with CBM, while the meta-analysis included 53 patients. Among the nine studies, six were retrospective cohort studies, and three were RCTs.

### Characteristics of the included studies

The sample sizes in the nine included studies ranged from 16 to 98. The mean age (± standard deviation) was 34.5 ± 12.7 years. Males comprised 75% of the patients, while females comprised 25%. The summaries of the included studies, their main findings, and the baseline characteristics of their populations are presented in Table 1.

**Table 1:** Baseline characteristics of included studies

Study and year	Study design	Type of drug	Total sample size	Age (Mean, SD)	Sex, male/female (*n*)	Dose of cannabis	Follow-up	Comorbidities (OCD, ADHD, Anxiety)	Main findings
Milosev,2019 [54]	Retrospective cohort	MC, dronabinol, nabiximols, and street cannabis	98	35.8 (13.1)	84/14	•MC [g/day]: 2.2 (2.39), •Dronabinol [mg/day]: 43.2 (68.32), •Nabiximols [puffs/day]: 10.6 (8.89)	Mean duration of treatment with CBM was 62.1 – 73.9 months (median = 41, range 1–336, n = 77)	•OCD = 44 •OCB = 35 •ADHD = 34 •Depression = 36 •anxiety disorders = 21 •sleep disorders = 36	•CBM might be a treatment option even in patients unsatisfied with established treatment strategies
Muller-Vahl, 2003 [23]	RCT	D9-THC	24	33 (11)	NA	D9-THC (gelatin capsules of 2.5 and 5.0 mg) then was titrated to a target dosage of 10.0 mg D9-THC	6 weeks	NA	•In patients suffering from TS, treatment with D9-THC causes neither acute nor long-term cognitive deficits
Müller-Vahl,2023 [48]	RCT	Nabiximols (sublingual Oro mucosal spray)	97	37.4 (14.3)	73/24	•Flexible dose ranging from 1 to 12 puffs/day (1 puff nabiximols = 100 μl spray including 2.7 mg THC and 2.5 mg cannabidiol)	4 weeks	•ADHD = 14 •OCD = 17 •Anxiety = 41	• The number of responders in the nabiximols group was much larger compared to the placebo group
Anis,2022 [51]	Cohort study	MC	18	32.75 (8.23)	11/7	•MC average monthly dose (g):16.8—5.4 •MC use times per day: 2.7–0.8 •Quantity in each use (puffs/drops): 7.8—5.4	12 weeks	•ADHD = 14 •OCS = 12 • depressive episodes = 7 •Anxiety = 10	•It is suggested that MC might be a treatment option for resistant TS patients, and MC has a significant effect on tics, premonitory urges, and patients' overall quality of life
Abi-Jaoude,2017 [50]	Cohort study	Cannabis was used in many forms: smoked, vaporized, and ingested with food	19	32 (12.3)	16/3	•The estimated average daily dose varied substantially, from less than 0.1 g to 10 g, for a median of 1 g daily	NA	•OCD = 13 •ADHD = 11 •Anxiety = 3	• cannabis seems to be a promising treatment option for tics and associated symptoms
Thalera,2018 [28]	Retrospective cohort	MC	42	34.45 (11.84)	33/9	•29.37 (9.48) g	NA	•OCD = 27 •ADHD = 26 •Depression = 15 •Anxiety = 20	•The use of MC shows potential in the treatment of GTS as it has shown to be highly satisfactory for most patients
Müller-Vahl,1998 [56]	Retrospective cohort	Marijuana	17	30.5 (8.87)	15/2	•Of the 17 patients, 2 patients had regularly smoked for a longer period (> 1 year) and 15 patients reported occasional use of marijuana	NA	•OCS = 17 •ADHD = 10	•The study results further support the idea that marijuana has a positive impact on reducing tics and behavioral disorders in individuals with Tourette syndrome
Bloch,2021 [49]	Phase 2 pilot study	D9-THC, THX-110	16	35 (13)	10/6	•The THX-110 (maximum daily D9-THC dose, 10 mg, and a constant 800 mg dose of PEA)	6 months	•OCD = 12 •ADHD = 3 •Anxiety = 5 •Depression = 6	• THX-110 treatment led to an average improvement in tic symptoms of more than 20%
Barchel,2022 (52)	Cohort study	D9-THC and cannabidiol	70	Median age 31	47/10	• D9-THC, 123 mg and cannabidiol 50.5 mg	6 months	•OCD = 9 •anxiety disorder = 19	• A statistically significant improvement was identified in quality of life and in reducing the number of medications after tetrahydrocannabinol

*ADHD* attention deficit hyperactive disorder, *CBM* cannabis-based medicine, *D9-THC or THC* tetrahydrocannabinol, *g-gram*, *GTS* Gilles de la Tourette’s syndrome, *MC* medicinal cannabis, *NA* not applicable, *OCD* obsessive–compulsive disorder, *RCT* randomized controlled trial, *SD* standard deviation, *THX-110* combination drug of THC and palmitoylethanolamide

### Quality assessment

**Fig. 2:**
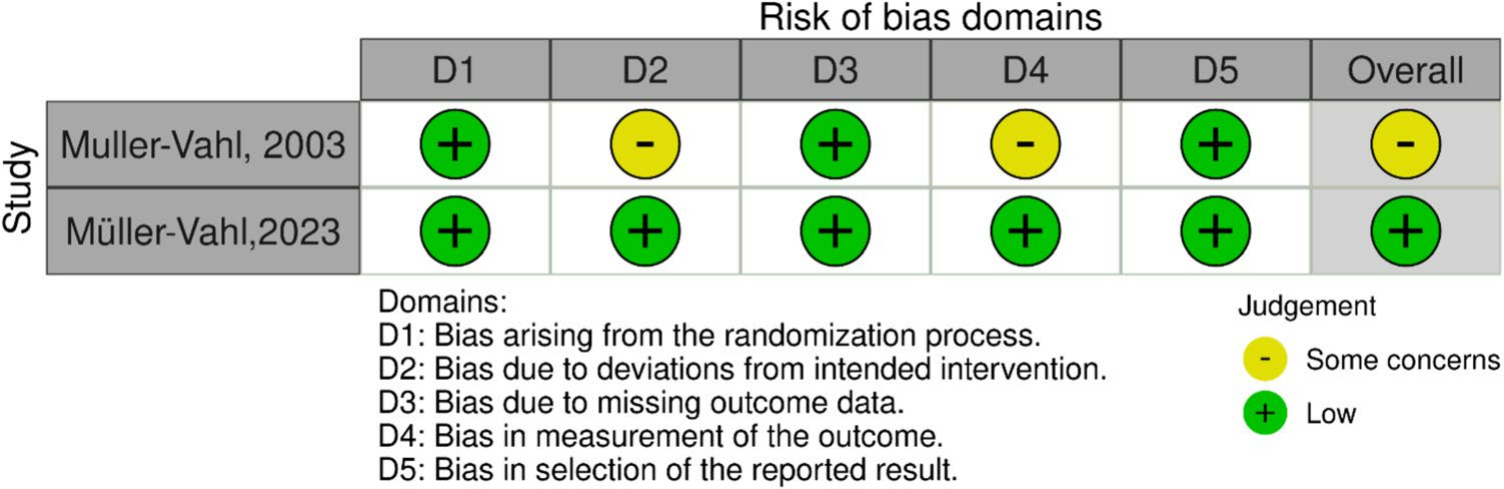
Quality assessment of RCT using ROB2 tool

For the RCTs, the RoB 2 tool showed that one study [48] was rated as having a low risk of bias across all domains, indicating a robust methodological approach. However, another study [23] raised some concerns and received an unclear rating in at least one domain (Fig. 2). Notably, a study by Bloch [49] had phase 2 RCTs. Hence, no quality assessment was conducted for it.

In terms of the Newcastle–Ottawa Scale (NOS) for cohort studies, four studies were rated as high quality (with a total score ranging from 7 to 9 stars), and two studies were rated as moderate quality (with a total score of six stars) (Table 2).

**Table 2:** Quality assessment of cohort studies using NOS scale

Study ID	Selection (max 4)	Comparability (max 2)	Outcome (max 3)	Total (max 9)
Milosev, 2019	☆☆☆	☆	☆☆	☆☆☆☆☆☆
Anis, 2022	☆☆☆☆	☆☆	☆☆	☆☆☆☆☆☆☆☆
Abi-Jaoude, 2017	☆☆☆	☆	☆☆☆	☆☆☆☆☆☆☆
Thalera, 2018	☆☆☆☆	☆☆	☆☆☆	☆☆☆☆☆☆☆☆☆
Vahl KR, 1998	☆☆☆	☆	☆☆	☆☆☆☆☆☆
Barchel, 2022	☆☆☆☆	☆☆	☆☆	☆☆☆☆☆☆☆☆

### YGTSS

**Fig. 3:**
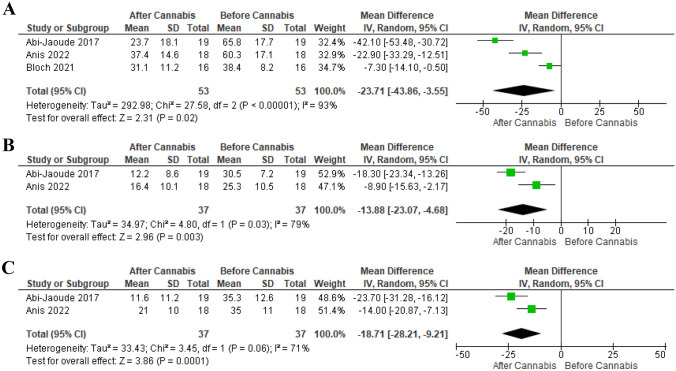
Analysis of the mean difference of YGTSS-TS score before and after cannabis

Three studies involving 53 patients utilized the YGTSS scale for outcome measurement. There was a significant reduction in the total YGTSS scores at the endpoint compared to the baseline (MD -23.71, 95% CI [-43.86 to -3.55], *P* = 0.02). Two RCTs involving 37 patients evaluated YGTSS-tic severity. There was a significant reduction in the tic severity score at the endpoint compared to the baseline (MD -13.88, 95% CI [-23.07 to -4.68], *P* = 0.003). A significant difference was also observed in the YGTSS-impairment (MD -18.71, 95% CI [-28.21 to -9.21], *P* < 0.001). Significant heterogeneity was found across the three outcomes (Fig. 3A, B, and C).

Sensitivity analysis was employed to address this heterogeneity in the YGTSS-total outcome. However, significant heterogeneity persisted even after removing any of the studies by Abi-Jaoude [50], Anis [51], or Bloch [49]. The reported heterogeneity was (*P* = 0.01, I-square = 84%), (*P* < 0.001, I-square = 96%), and (*P* = 0.01, I-square = 83%) respectively (Supplementary Fig. 1).

### PUTS and Y-BOCS

**Fig. 4:**
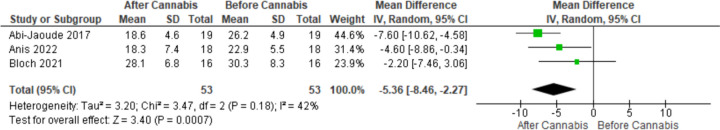
Analysis of the mean difference of PUTS score before and after cannabis

**Fig. 5:**
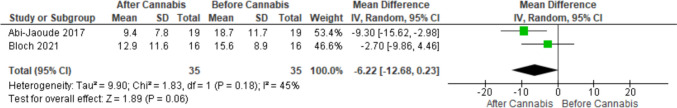
Analysis of the mean difference of Y-BOCS score before and after cannabis

Three studies involving 53 patients utilized the PUTS score as an outcome measurement. A significant difference was observed in the reduction of the PUTS score at the endpoint compared to the baseline (MD -5.36, 95% CI [-8.46 to -2.27], *P* = 0.0007). No heterogeneity was found among the pooled studies (*P* = 0.18, I-square = 42%) (Fig. 4). Sensitivity analysis was conducted by removing each of the studies by Abi-Jaoude [50], Anis, or Bloch [49]. The results remained significant (*P* = 0.03, *P* = 0.04, *P* < 0.001), and the reported heterogeneity varied (*P* = 0.49, *I*-square = 0%; *P* = 0.08, *I*-square = 67%; *P* = 0.26, *I*-square = 21%) respectively (Supplementary Fig. 2). Two studies involving 35 patients tested the Y-BOCS score. No significant difference was found in the reduction of the Y-BOCS score (MD -6.22, 95% CI [-12.68 to 0.23], *P* = 0.06) (Fig. 5).

## Discussion

In our systematic review and meta-analysis, we evaluated the efficacy of cannabis in treating TS. We focus on tic severity, premonitory urges, and OCD. We analyzed data from nine studies involving 401 patients with TS and included three in our quantitative synthesis involving 53 patients with TS, and we found significant reductions in tic severity and premonitory urges, as indicated by YGTSS and PUTS scores. However, our findings did not significantly impact obsessive–compulsive symptoms measured by the Y-BOCS.

TS poses significant challenges for patients and healthcare providers [3]. Despite the use of various agents to reduce the frequency and severity of TS-related tics and improve the patient's quality of life, there is a lack of high-quality evidence supporting their efficacy [3]. Only three agents—haloperidol, pimozide, and aripiprazole—have been approved by the Food and Drug Administration (FDA) for tic control [3, 14]. Nevertheless, due to the absence of universal treatment, many agents, including CBM, have been suggested for tic control.

Self-treatment with CBM by some patients has made understanding the use of such substances in TS more convenient. Although numerous studies have attempted to demonstrate the impact of CBM on the severity and frequency of tics, no definitive conclusions or recommendations have emerged from the available studies [23, 28, 48–56]. An early Cochrane review, which included only two RCTs involving 28 adults, could not endorse the use of CBM for tic control due to methodological limitations, small sample size, and the limited number of studies [57].

The pooled analysis of the included studies revealed significant reductions in the total YGTSS score, YGTSS-tic severity, and YGTSS-impairment among the CBM group at the endpoint compared to the baseline. These findings necessitate a confirmatory study that includes a comparison with a placebo. Notably, significant heterogeneity was detected among studies reporting the total YGTSS score and YGTSS-tic severity score, which the sensitivity analysis could not resolve. Apart from the differences in methodologies and study designs among the studies, *Cannabis sativa* contains more than 60 types of CBM, 43 of which have varying strengths and concentrations. This leads to a heterogeneous composition of test drugs, challenging comparative studies and potentially contributing to the observed pooled heterogeneity [3, 57].

Most patients with TS experience an uncomfortable period of either sensory sensations (e.g., itching or pressure) or mental phenomena (e.g., a feeling that something is not quite right) preceding their tics. These are known as premonitory urges. These urges are often more distressing and embarrassing than the tics themselves. As such, they are a target for TS therapies, especially behavioral therapy, as it may facilitate the suppression of the impending tics. The PUTS scale is the most frequently used self-report measure to assess the severity of premonitory urges [44, 58]. Our analysis showed a significant reduction in PUTS scores after administering CBM compared to baseline. There was insignificant heterogeneity among the studies, which further supports the potential of CBM in controlling tics and premonitory urges.

The disability suffered by patients with TS is not only attributed to the tics but also to the co-existence of other psychiatric comorbidities such as ADHD and OCD symptoms. Y-BOCS assessing the clinical severity of obsessive–compulsive symptoms has been used in many studies to evaluate the extent of such symptoms among patients with TS [59, 60]. Notably, the present analysis revealed that CBM reduces the Y-BOCS scores compared to baseline; however, this finding was deemed insignificant.

## Strength and limitations

To the best of our knowledge, this is the first systematic review and meta-analysis evaluating the effectiveness of CBM among patients with TS using various scales. However, the present study does have certain limitations. Despite including clinical trials and cohort studies, the number of studies and patients in the quantitative analysis was small, raising concerns about the study results’ generalizability. Due to the limitations and few available studies, we could not compare the CBM group with placebo (or other drug) groups. Furthermore, heterogeneity was observed in some outcomes despite conducting a sensitivity analysis.

## Conclusions

The present study suggests favorable and potentially effective results with CBM in reducing the severity of tics and premonitory urges. Large studies with rigorous methodologies, unified drug components, and fixed doses are needed to estimate their effectiveness accurately.

## Supplementary Information

Below is the link to the electronic supplementary material.

Supplementary file1 (DOCX 38 KB)

Supplementary file2 (DOCX 39 KB)

## Data Availability

All data generated or analyzed during this study are included in this published article.
