# Supplementary material for: Efficacy of cannabis-based medicine in the treatment of Tourette syndrome: a systematic review and meta-analysis
Source: Eur J Clin Pharmacol. 2024 Jul 10;80(10):1483–93. doi: 10.1007/s00228-024-03710-9 (PMC11393157; doi:10.1007/s00228-024-03710-9)
Supplement: Supplementary file 1 — Supplementary file1 (DOCX 38 KB) [file 228_2024_3710_MOESM1_ESM.docx]

## YGTSS-total

##
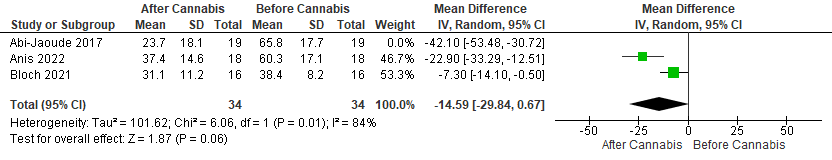


Heterogeneity after removal of Abi-Jaoude 2017

#
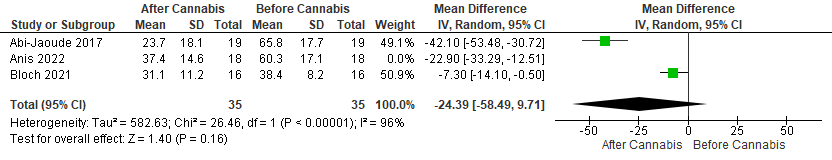


# Heterogeneity after removal of Anis 2022
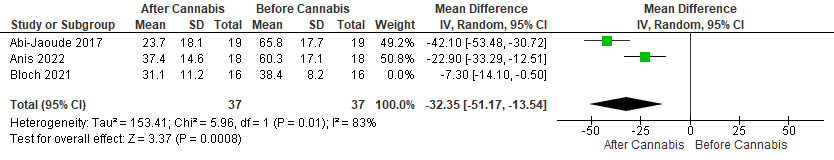


Heterogeneity after removal of Bloch 2021
