# Supplementary material for: Efficacy of cannabis-based medicine in the treatment of Tourette syndrome: a systematic review and meta-analysis
Source: Eur J Clin Pharmacol. 2024 Jul 10;80(10):1483–93. doi: 10.1007/s00228-024-03710-9 (PMC11393157; doi:10.1007/s00228-024-03710-9)
Supplement: Supplementary file 2 — Supplementary file2 (DOCX 39 KB) [file 228_2024_3710_MOESM2_ESM.docx]

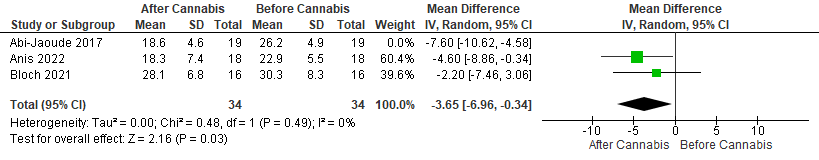


Analysis after removal of Abi-Jaoude 2017


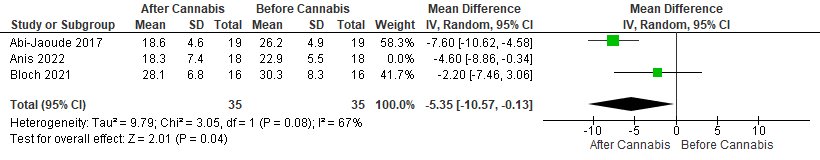


Analysis after removal of Anis 2022


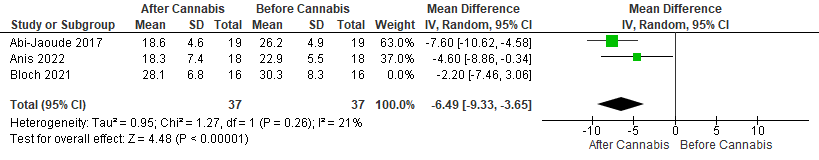


Analysis after removal of Bloch 2021
